# Hybrid teaching after COVID-19: advantages, challenges and optimization strategies

**DOI:** 10.1186/s12909-024-05745-z

**Published:** 2024-07-12

**Authors:** Xiaoran Wang, Jiangheng Liu, Shuwei Jia, Chunmei Hou, Runsheng Jiao, Yan Yan, Tengchuang Ma, Ying Zhang, Yanyan Liu, Haixia Wen, Yu-Feng Wang, Hui Zhu, Xiao-Yu Liu

**Affiliations:** 1 School of Basic Medical Sciences, Harbin Medical University, Harbin, Heilongjiang 150081 China; 2 Harbin Medical University Cancer Hospital, Harbin, Heilongjiang 150086 China; 3 International Translational Neuroscience Research Institute, Zhejiang Chinese Medical University, Hangzhou, 310053 China

**Keywords:** Hybrid teaching, Online teaching, Physiology education, Teaching model

## Abstract

**Background:**

In the post-pandemic era of higher education, hybrid teaching has emerged as a prevalent approach and is anticipated to persist as a defining trend in the future teaching reforms worldwide. However, despite its widespread adoption, certain limitations have become apparent. The objective of this study is to identify the genuine factors that impact students’ performance, explore strategies that teachers can employ to enhance their teaching effectiveness and enhance students’ academic self-efficacy.

**Methods:**

The study was performed among undergraduate medical students enrolled in Physiology course at Harbin Medical University in 2020 and 2022. Since 2020, influenced by the COVID-19 pandemic, a hybrid teaching method based on an established offline teaching model called BOPPPS was implemented. A questionnaire was performed in both 2020 and 2022 to evaluate students’ satisfaction and efficiency of our hybrid teaching. A comparison was also carried out on the final examination scores of students majoring in Pharmacy and Clinical Pharmacy across the years 2020 to 2022.

**Results:**

The final examination scores of students in 2022 were significantly lower than those in 2020 and 2021 both in Pharmacy and Clinical Pharmacy majors. There was also a decrease of the score in students of Clinical Pharmacy in 2021 compared to 2020. The questionnaire indicated that over half (52.0%) of the students in 2022 preferred offline teaching method, in contrast to 39.1% in 2020. There were obvious changes in students from 2020 to 2022 about the disadvantages of hybrid teaching, the improvement of students’ learning ability and the duration of students’ autonomous learning. Through cross statistical analysis, online learning styles, learning ability improvement and students’ learning burden have been identified as the primary factors influencing their preference for future teaching method.

**Conclusions:**

Hybrid teaching is still a necessary trend in the future teaching reform base on its multiple advantages. However, in order to improve the teaching outcomes and foster students’ participation and learning initiatives, it is imperative to undertake additional reforms in the future teaching process.

**Supplementary Information:**

The online version contains supplementary material available at 10.1186/s12909-024-05745-z.

## Introduction

The adoption of hybrid teaching, characterized as a combined approach encompassing both online and face-to-face instructional methods, has proliferated globally during the pandemic of COVID-19. Although digital teaching has existed for decades, its widespread adoption during this crisis was unprecedented due to various advantages such as location flexibility, convenient lecture recording, efficient communicating and prompting feedback mechanisms [1–3]. Multiple studies have found that hybrid teaching method supported by learning management systems and innovative features like online quizzes, can be as effective as traditional face-to-face classes, particularly in fostering independent and autonomous learning [4, 5].

However, along with the wide application of hybrid teaching, challenges have emerged in the form of heightened self-motivational demands, reliance on consistent internet access, and health concerns related to screen-induced eyestrain [6]. Moreover, researchers have found that hybrid teaching cannot significantly improve student engagement in academic activities in China and USA [7]. Furthermore, the normalization of the COVID-19 epidemic has been found to affect students’ mental health which is positively correlated with learning burnout [8]. A study in China has found that the prevalence of academic burnout among nursing students stands at 31.5% and students with low academic self-efficacy are more susceptible to learning burnout [9]. Given the inevitability of hybrid teaching in the post-pandemic era, it is necessary to figure out what teachers can do to enhance hybrid teaching effectiveness and students’ academic self-efficacy.

Physiology, a cornerstone of medical science, provides a basic understanding of healthy human body functions and plays an important role as a link between preclinical courses and subsequent clinical courses [10]. Amidst the COVID-19, a hybrid approach was implemented for the Physiology curriculum in our university and we received many good comments from students [11]. Nevertheless, with the in-depth development of hybrid teaching, some complaints from students have gradually emerged. Therefore, this study aims to investigate students’ preferences towards hybrid teaching, conduct an in-depth analysis of factors influencing these preferences and propose strategies for improving instructional methods and enhancing students’ academic self-efficacy.

## Methods

### Ethics statement

This study was approved by the Department of Physiology at Harbin Medical University. The procedures of this study adhered to the guidelines of the Declaration of Helsinki. This project was deemed non-human-subjects research by the Institutional Review Board of the Harbin Medical University according to “ethical review measures for life sciences and medical research involving human beings” (Order No. 11 of the National Health and Family Planning Commission of China, December 2016). Due to the online survey approach, the written informed consent could not be received. Therefore, verbal informed consent for survey was approved by the Ethics Committee of the Institutional Review Board of Harbin Medical University and obtained from each participate. All data collected from the participants were kept anonymous to protect their privacy.

### Study subjects

This study involved undergraduate medical students who were part of the cohorts beginning in 2019 and 2021, and subsequently participated in the Physiology course offered at Harbin Medical University during the first semesters of 2020 and 2022, respectively. The students were enrolled in the majors of Pharmacy (a four-year program), Clinical Pharmacy (a five-year program) and Basic Medicine along with Clinical Medicine (seven /eight-year long-term systems) and they all had received systematic pre-college education under the same guideline and passed the requirements of entrance examination. In the Physiology learning, all participants had received standardized instructional methodologies from the faculty of Physiology Department. Notably, the emergence of COVID-19 in 2020 marked a significant shift in teaching modalities. For the majority of students, this was their first exposure to online teaching. Contrastingly, by 2022, the students had prior experience with online learning, either during high school education or during their initial semester at the university journey. An anonymous questionnaire was distributed to evaluate student perceptions of our hybrid teaching modality. We aim to utilize this feedback to enhance the teaching method and improve the teaching effectiveness.

### Hybrid teaching method

BOPPPS, standing for Bridge-in, Objective, Pre-assessment, Participatory learning, Post-assessment and Summary, is a widely used offline teaching model. HBOPPPS teaching modality, an innovative hybrid teaching method introduced in 2019, cleverly incorporates online instructional techniques into the BOPPPS framework [11]. In the process of promoting HBOPPPS hybrid teaching model, our team constructed an online learning resource for students, including course-associated micro-lecture videos (85 in total, 5 ∼ 10 min/video) based on the textbook of Physiology (Ting-Huai Wang, People’s Medical Publishing House, 9th Ed), course-associated science stories, lectures delivered by renowned doctors, virtual simulation experiments and chapter tests (https://www.xueyinonline.com/detail/235823098).

Prior to the class, students were provided with a comprehensive course guidance through the “Xuexi Tong” mobile application. This guidance encompassed an introduction to the course objectives, the key knowledge to be mastered, and the goals for fostering abilities. Subsequently, during and following the lecture, students had access to a variety of interactive elements through the online application, including sign-in procedures, multi-choice questions, quick response questions, task allocation and summaries.

### Data collection

Prior to data collection, a power analysis was conducted to determine an appropriate sample size that would provide adequate statistical power to detect meaningful differences in our study outcomes. Based on the power analysis, we aimed to collect data from at least 158 total participants to achieve a power of 0.8 with an alpha level of 0.05. A total of 128 and 200 valid questionnaires were collected in 2020 and 2022 respectively from students enrolled in four different majors (Table 1). In order to evaluate the relative efficacy of our teaching methods, we collected the final exam scores of Pharmacy and Clinical Pharmacy students who were the main participants in the questionnaires. All data was available in the supplemental file 1.

**Table 1:** Basic Condition Statistics of Students in the survey year 2020 and 2022

Survey Year		2020	2022
Number of students		128	200
Gender	Male	47	65
	Female	81	135
Program of education	Four-year	38	79
	Five-year	43	74
	Long-term system	47	47

### Data analysis

All analysis was performed using SigmaStat program (SPSS 19, Chicago, IL). Comparisons of the final scores between different years were performed using one-way ANOVA followed by Bonferroni or Dunnett T3 test, as appropriate. After the completion of data collection and the subsequent statistical analysis, effect sizes (ES) were reported to facilitate the interpretation of the findings. Utilizing the free software G*Power 3.1 [12], the ES values were calculated to ensure that our study was sufficiently powered (1 − β = 0.8) to detect significant differences (α = 0.05) among the analyzed variables. Comparative analysis of proportions was executed utilizing the chi-square test. Data are expressed, as mean ± SEM and *P* < 0.05 was considered statistically significant.

## Results

### Student preferences for future teaching modalities

By 2022, hybrid-teaching method, which combined online and offline teaching, had been employed in Physiology teaching at Harbin Medical University for about 4 years for the reason of COVID-19 pandemic. In the process of implementing hybrid teaching, the effect of online teaching and the feedback from students on this modality has been in dispute. To gain a clearer understanding of students’ true opinions, questionnaires were performed in both 2020 and 2022.

**Fig. 1:**
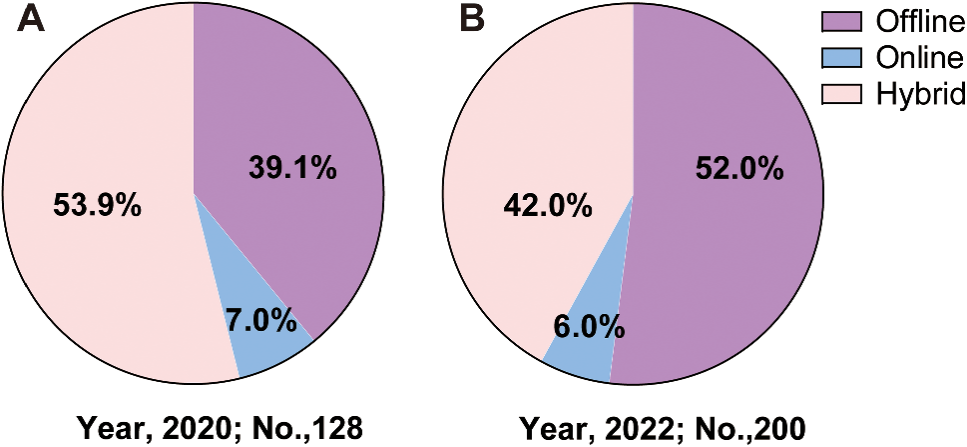
Choices of students for their favorite teaching methods. **A**. Students from 2020. **B**. Students from 2022. No, number of students

First, we compared students’ preferences for Physiology teaching methods post-pandemic. Beyond our expectation, the preference of choosing online teaching remained relatively unchanged, while the rate of choosing hybrid teaching method decreased largely from 2020 to 2022, albeit without reaching statistical significance (53.9% in 2020 vs. 42.0% in 2022, *P* = 0.07) (Fig. 1). This result suggested that students may not be fully satisfied with the hybrid teaching approach. It was imperative for us to delve deeply into the reasons behind this dissatisfaction.

### Students’ scores in the past three years

**Fig. 2:**
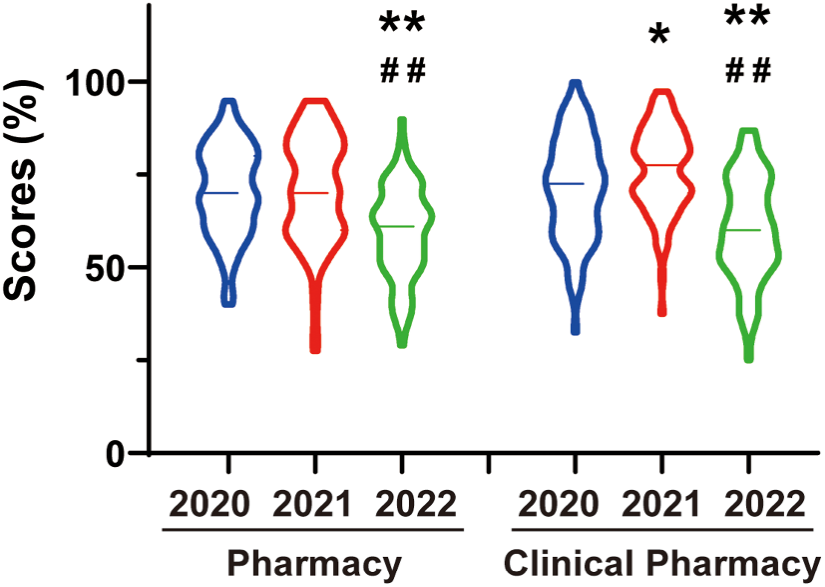
Scores of students’ final examination in the past three years. *P < 0.05, ** *P* < 0.01, compared to 2020; ## *P* < 0.01 compared to 2021

Generally, final exam score is a crucial metric for assessing teaching efficiency. To evaluate the impact of Hybrid teaching method, final exam scores were compared among the past 3 years (2020 ∼ 2022) from four-year and five-year students. The final exam was composed of two parts, i.e. subjective and objective questions. However, the final examination of 2022 had to be performed on a mobile App and it was only consisted of single-choice questions (i.e., objective questions). This modification was carried out as a national precautionary measure in response to the ongoing preventative measures against COVID-19. Therefore, we compared the objective scores of students major in Pharmacy and Clinical pharmacy separately from 2020 to 2022 as a representative. Our results showed that the average score of students in 2022 decreased significantly compared to those in 2020 and 2021, both in Pharmacy and Clinical pharmacy students (Pharmacy: 70.7 ± 1.27% in 2020, *n* = 92; 70.9 ± 1.58% in 2021, *n* = 85; 59.5 ± 1.28% in 2022, *n* = 94, ES = 0.591, *P* < 0.01 in ANOVA; Clinical pharmacy: 71.2 ± 1.5% in 2020, *n* = 86; 76.2 ± 1.23% in 2021, *n* = 88; 60.3 ± 1.47% in 2022, *n* = 88, ES = 0.596, *P* < 0.01 in ANOVA, see Fig. 2). This observed decline in academic performance may indicate the teaching effect gradually decreased with the extension of hybrid teaching time.

### Advantages and disadvantages of online teaching

**Fig. 3:**
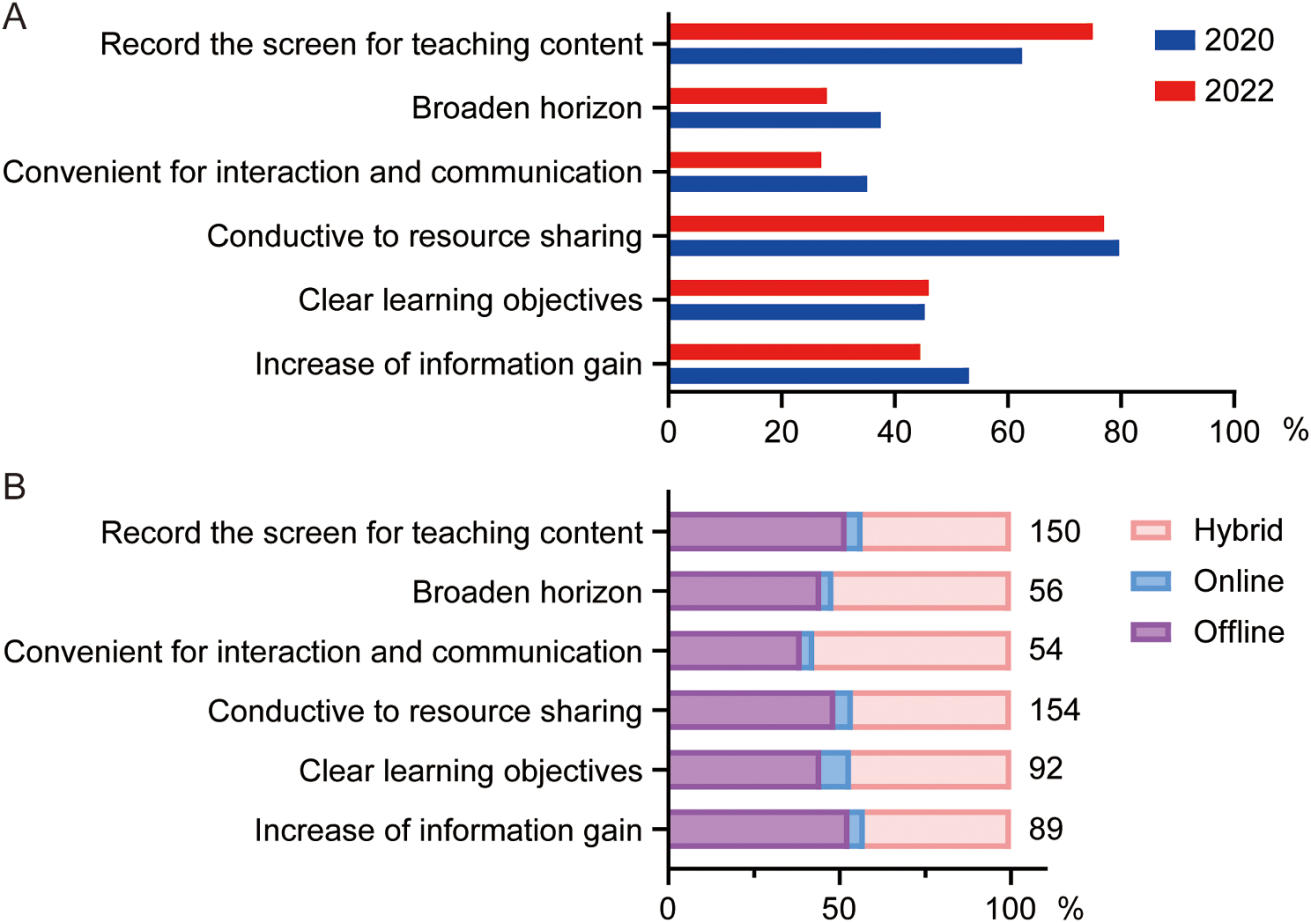
Effect of advantages on the choice of students. **A**. Advantages of online teaching suggested by students of 2020 and 2022. **B**. Crossover statistics illustrating the relationship between students’ preferences for teaching methods and their perceived advantages in 2022. The numbers next to the bar chart represent the number of students participating

To figure out why students’ confidence in hybrid teaching gradually waned over the years, we analyzed related data from questionnaires from 2020 to 2022. Our results showed an obvious increase in the ratio of students thinking that it was convenience for recording the teaching content (62.5% in 2020 vs. 75.0% in 2022, Fig. 3A), despite insignificant differences in evaluation advantages of hybrid teaching. Besides, there were also decreases in “Broaden horizon (37.5% in 2020 vs. 28.0% in 2022)”, “Convenient for interaction and communication (35.2% in 2020 vs. 27.0% in 2022)” and “Increase of information gain (53.1% in 2020 vs. 44.5% in 2022)” (Fig. 3A).

To find out whether the advantages can effect students’ choices, we conducted a crossover statistic which can illustrate the relationship between students’ preferences for teaching methods and their perceived advantages (Fig. 3B). While the results did not reveal any significant statistical differences, a notable trend emerged that students who thought it was convenient for interaction and communication preferred hybrid teaching method.

**Fig. 4:**
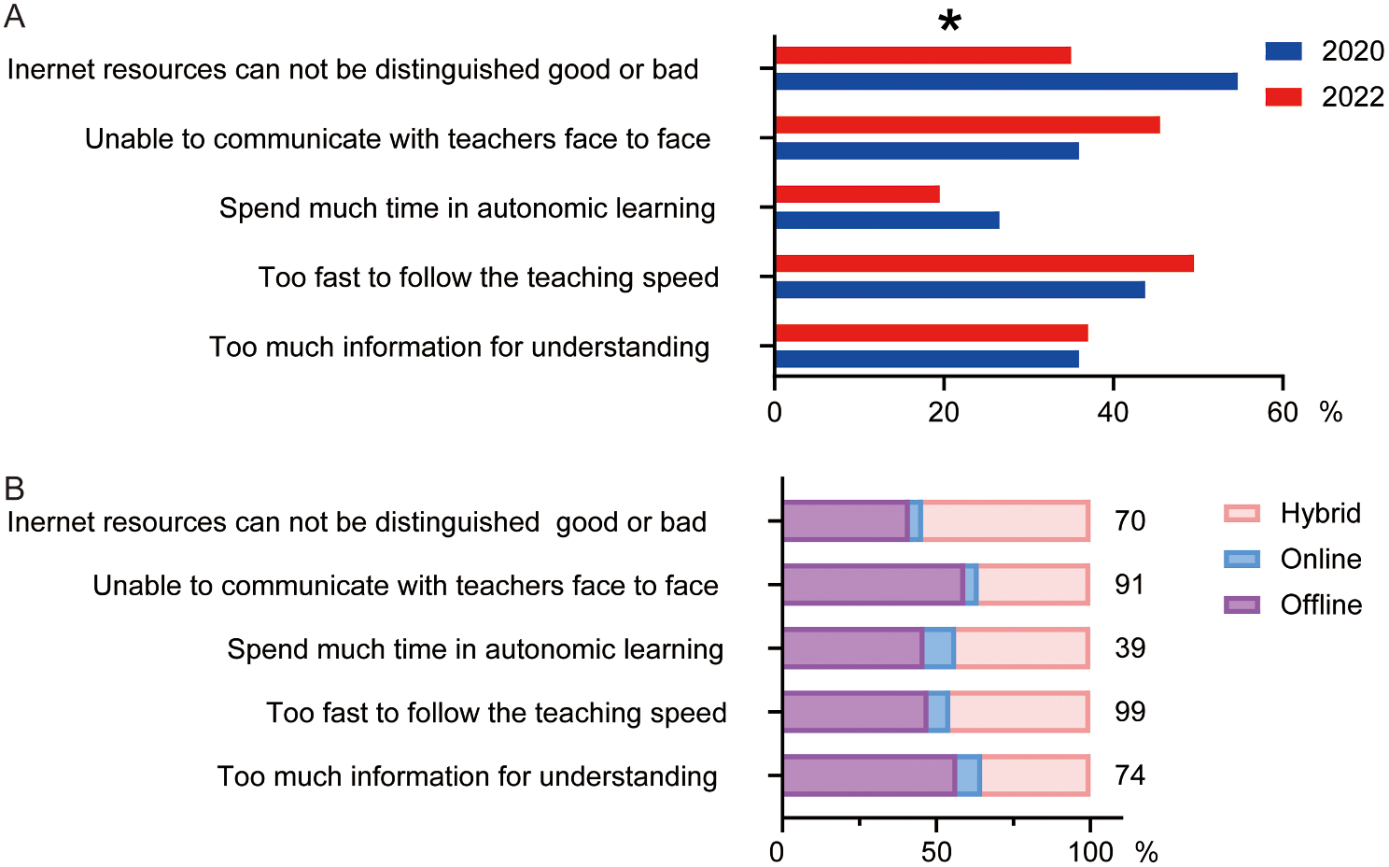
Effect of disadvantages on the choice of students. **A**. Disadvantages of online teaching suggested by students of 2020 and 2022. **B**. Crossover statistics illustrating the relationship between students’ preferences for teaching methods and their perceived disadvantages in 2022. ES = 0.138, *P < 0.05, comparison among all groups by Chi-squared test for R×C table. The numbers next to the bar chart represent the number of students participating

Analysis of disadvantages about hybrid teaching revealed significant differences between the results of 2020 and 2022. There was an obvious decrease in the option of “Internet resources cannot be distinguished good or bad” (54.7% in 2020 vs. 35% in 2022, see Fig. 4A) and students in 2022 who chose this option tended to choose hybrid teaching method (41.4% in offline, 4.3% in online and 54.2% in hybrid teaching, see Fig. 4B). There was also a clear increase in the option of “Unable to communicate with teachers face to face” (35.9% in 2020 vs. 45.5% in 2022, see Fig. 4A) and students in 2022 who chose this option tended to choose offline teaching method (54.0% in offline, 4.0% in online and 33.0% in hybrid teaching, see Fig. 4B). These results highlight the crucial role of face to face communication with teachers in influencing students’ choices.

### Fondness for styles of hybrid teaching

**Fig. 5:**
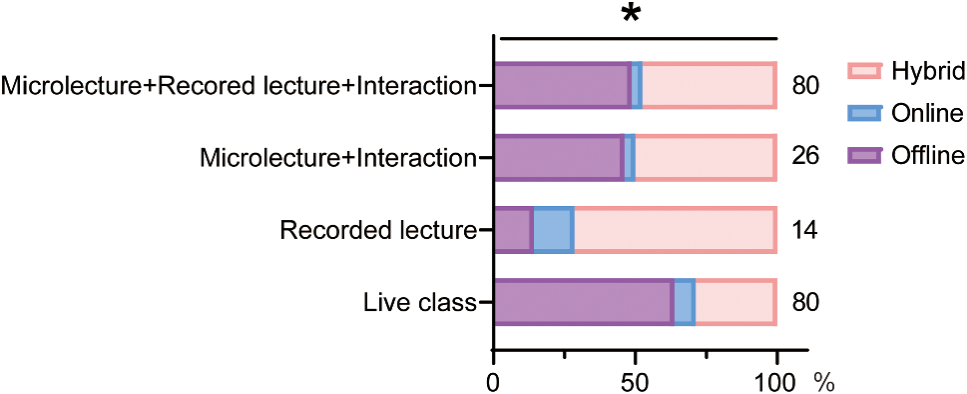
Crossover Statistics illustrating the preferences for different hybrid learning styles of students in 2022 and their preferences for the three teaching methods. ES = 0.261, *P < 0.05, comparison among all groups by Chi-squared test for R×C table. The numbers next to the bar chart represent the number of students participating

In 2020 to 2022, the COVID-19 pandemic had significantly influenced the teaching styles adopted in our courses, resulting in various hybrid teaching approaches. We surveyed the relationship between different hybrid teaching styles and the choice of learning method in students of 2022. The data revealed that most of the students who liked live class tended to choose offline teaching method (63.8%) and students who liked recorded lectures preferred hybrid teaching method (71.4%). The remaining students showed similar preferences for hybrid and offline teaching (Fig. 5).

### Improvement of learning ability

**Fig. 6:**
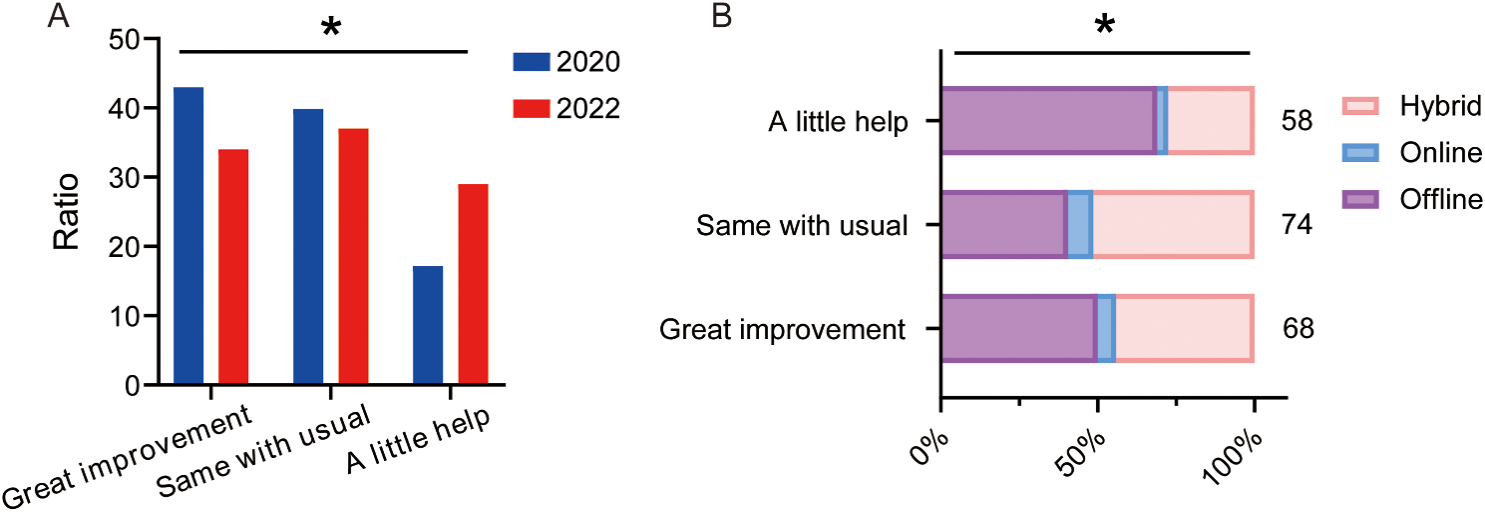
Effect of learning ability improvement on the choice of students. **A**. Improvements of learning ability suggested by students of 2020 and 2022. **B**. Crossover statistics illustrating the relationship between students’ preferences for teaching methods and improvements of learning ability in 2022. ES = 0.171; ES = 0.244, *P < 0.05, comparison among all groups by Chi-squared test for R×C table. The numbers next to the bar chart represent the number of students participating

Since the hybrid teaching method was designed to improve students’ autonomous learning ability, we assessed effects of our hybrid teaching on their learning ability. Unfortunately, the result revealed a significant increase in the proportion of students who thought that hybrid teaching didn’t help improving their learning ability (17.2% in 2020 vs. 29.0% in 2022) and a decrease in the ratio of students who thought that hybrid teaching improved autonomous learning ability greatly (43.0% in 2020 vs. 34.0% in 2022)(Fig. 6A). There was also a significant difference in the cross-analysis. Students who thought hybrid teaching didn’t help improving learning ability preferred offline teaching compared to students who thought hybrid teaching help improving learning ability (69.0% in students who thought a little help, 40.5% in students who thought same with usual and 50.0% in students who thought great improvement, *P* < 0.05 in Fisher’s Exact Test, see Fig. 6B).

### Duration of autonomous learning

**Fig. 7:**
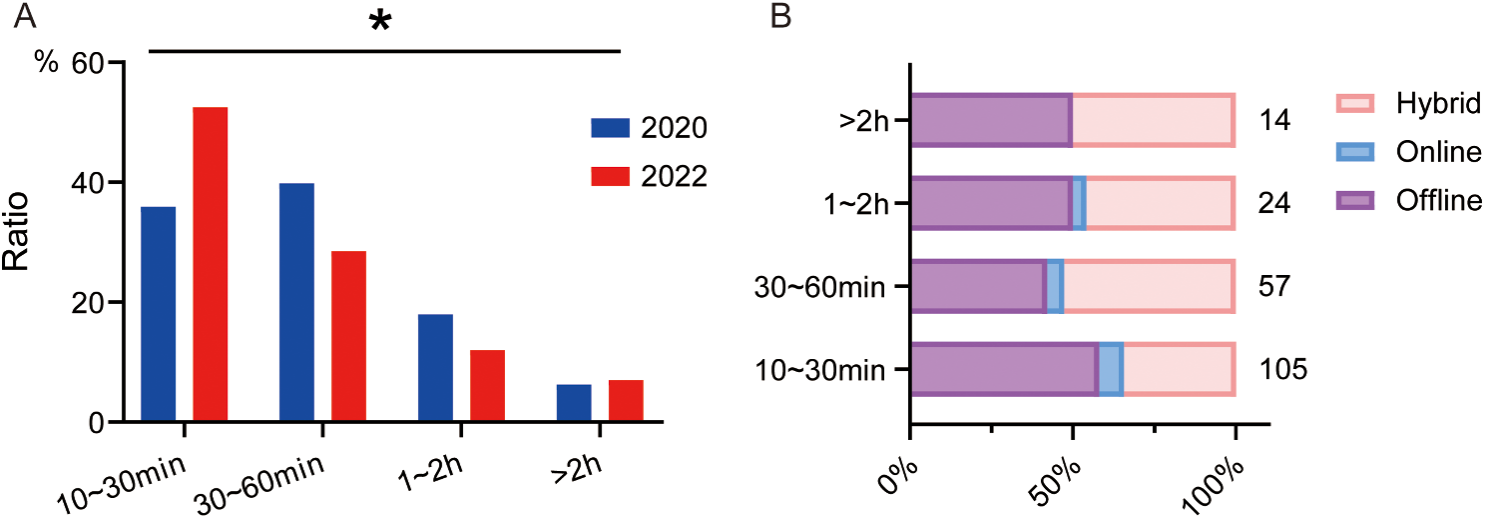
Effect of autonomous learning duration on the choice of students. **A**. Duration of autonomous learning suggested by students of 2020 and 2022. **B**. Crossover statistics illustrating the relationship between students’ preferences for teaching methods and autonomous learning duration in 2022. ES = 0.182, *P < 0.05, comparison among all groups by Chi-squared test for R×C table. The numbers next to the bar chart represent the number of students participating

Upon the survey of 2020 and 2022, there was a significant difference in the duration of students’ autonomous learning. The ratio of students spending 10 ∼ 30 min in autonomous learning increased in 2022 (35.9% in 2020 vs. 52.5% in 2022) and ratios of students spending 30 ∼ 60 min and 1 ∼ 2 h decreased (Fig. 7A). Although there was no difference between learning duration and students’ choices in the cross-analysis, it was noteworthy that student spending 30 ∼ 60 min on autonomous learning preferred hybrid teaching method, while student spending 10 ∼ 30 min on autonomous learning tended to prefer offline teaching method (Fig. 7B).

### Burden of learning

**Fig. 8:**
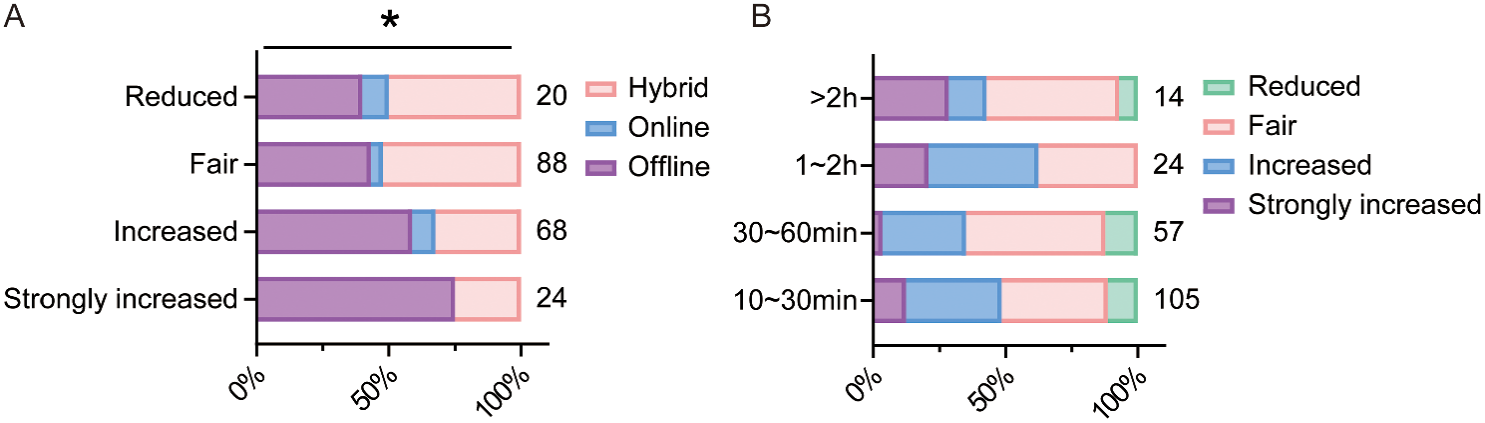
Effect of learning burden on the choice of students in 2022. **A**. Crossover statistics illustrating the relationship between students’ preferences for teaching methods and their learning burden. **B**. Relationship between autonomous learning duration and learning burden. ES = 0.261, *P < 0.05, comparison among all groups by Chi-squared test for R×C table. The numbers next to the bar chart represent the number of students participating

Burden of learning refers to the combination of workload, stress levels, and overall demands experienced by students during the learning process, which is a crucial factor influencing students’ preferences for teaching methods [13]. A high symptom burden from the acute stress response according to the COVID-19 pandemic is common among healthcare students [14]. We analyzed the relationship between learning burden and the choice of learning method in students of 2022. Our findings revealed that students who felt increased learning burden from hybrid teaching method preferred to choose offline teaching (Strongly increased, 75.0%; Increased 58.8%), while students who thought online teaching didn’t increase or reduce their learning burden preferred to choose hybrid teaching (Reduced, 50.0%; Fair, 52.3%) (Fig. 8A).

So where did the burden originate and whether the duration of autonomous learning played an important role? From the survey, we could see there was no significant difference between the duration of autonomous learning and learning burden. Unexpectedly, the smallest ratio of students choosing “hybrid teaching strongly increased learning burden” was among those who spent 30 ∼ 60 min in autonomous learning. This observation underscores that learning duration may not be the sole or decisive factor influencing the learning burden (Fig. 8B).

## Discussion

### Dropping of students’ scores

The COVID-19 pandemic has prompted most universities to adopt a hybrid teaching approach, which has generally been well-received [11, 15, 16]. However, our findings indicated a concerning decline in students’ performance, particularly in objective exam components, when assessments were conducted via a mobile application platform. This observation aligns with our previous work [11], which demonstrated that hybrid teaching can enhance performance in subjective exam sections. Since students surveyed in 2020 and 2022 were from different cohorts but received Physiology teaching from the same team and same method, this decline suggests that certain aspects of the hybrid teaching approach may require further refinement. The aim of our study was to explore these areas for potential improvement and to identify the factors contributing to the observed changes in performance, with the goal of enhancing the hybrid teaching model.

### Advantages and disadvantages of online teaching

The integration of online teaching into hybrid teaching has presented both opportunities and challenges. In the questionnaire, we surveyed the advantages and disadvantages of online teaching. Our survey findings revealed that while the perceived advantages remained relatively stable between 2020 and 2022, there was a notable increase trend in the number of students preferring recorded lectures. This preference aligns with the flexibility students seek to learn at their own pace, as evidenced by previous researches [17, 18]. Additionally, the convenience of sharing resources through online platforms was another advantage highlighted by our respondents. Despite these benefits, the preference for hybrid teaching was not uniformly increased, indicating that other factors might influence students’ preferences. Students who perceived online teaching as facilitating better interaction and communication, and as a means to broaden their perspectives, were more inclined towards hybrid learning. This suggests that while certain aspects of online teaching are valued, the integration into a hybrid model must be carefully considered to enhance its overall appeal and effectiveness.

Identifying the disadvantages of online teaching is crucial for improving hybrid teaching quality. The 2022 survey responses indicated a diminished concern regarding the quality of online educational resources compared to the perceptions in 2020. This shift in students’ attitude could imply a growing adaptability to online materials or a potential desensitization to the variability in resource quality. Interestingly, those students who acknowledged this limitation still preferred hybrid teaching, suggesting a deeper engagement with our hybrid teaching modality. Furthermore, two significant challenges faced by students in 2022 were identified “the teaching speed was too fast to follow” and “unable to communicate with teachers face to face”. These issues align with global trends observed in online education [16], where the absence of direct communication are frequently cited as drawbacks. The perception of a rapid teaching pace can be attributed to multiple factors, including the integration of multiple disciplines in hybrid teaching, fixed teaching hours of Physiology by school, and the varying abilities of students themselves. Addressing these specific issues is crucial to optimizing the hybrid teaching experience.

### Styles of online teaching

In the practical application, there are diverse styles of online teaching, including some related online micro lectures, live lectures and recorded lectures, which can be combined flexibly. The survey data from our study indicated a clear preference among students for different modalities of online instruction, which significantly influences their preference for different teaching methods. Students who favored live lectures, which offer a dynamic and interactive experience akin to in-person classes, were more inclined towards traditional offline teaching. Conversely, those who preferred recorded lectures, appreciated for their flexibility and ability to review material at any time, showed a greater preference for hybrid learning. This result was consistent with a research from Egypt where more than half of students (63%) agreed that online recorded video tutorials (e.g., YouTube) were the most effective form of online medical education [19]. Another group of students in 2022 who appreciated the combination of micro lectures, recorded lectures and face to face communications did not show obvious bias for either offline or hybrid teaching method [20]. These insights suggest that students’ engagement and satisfaction with hybrid teaching can be optimized by offering a varied and flexible approach to online instruction which are tailored to the content and needs of the course.

### Learning duration and burden

Our questionnaire data highlighted the impact of perceived learning burden on students’ preferences for teaching methods. Approximately half of the students reported that hybrid teaching heightened their learning burden, whereas the remaining half perceived either no significant impact or even a reduction in their burden. Interestingly and logically, students who perceived a reduction or no change in their learning burden due to hybrid teaching tended to prefer this teaching method. Conversely, those who experienced an augmented burden preferred traditional offline teaching.

The survey also explored the relationship between the duration of autonomous learning and perceived learning burden. It was found that more than half of the students in 2022 spent about 10 ∼ 30 min on autonomous learning and half of these students felt no change or even a reduction in the learning burden. Only students spending 1 ∼ 2 h on autonomous learning, accounting for 12% of the total in 2022, felt an increased learning burden. The reduced time spent on autonomous learning by students in 2022 might be attributed to the widespread implementation of hybrid teaching, which was time-consuming. These results suggests that the duration of self-directed study is not directly proportional to the perceived learning burden, and other factors, such as the learning environment and the student’s mindset, may exert a greater influence.

The influence of learning burden on the choice of teaching method underscores the need for a nuanced approach to curriculum design. While it is tempting to consider reducing homework or learning difficulty to alleviate students’ burden, it is essential to balance this against the potential benefits of challenging work in enhancing learning abilities. This balance is a topic that warrants further exploration and discussion.

### Learning ability

The purpose of teaching reform is to encourage active learning rather than passively receiving knowledge. Our survey data indicated that the majority of students reported an enhancement in their learning ability as a result of the hybrid teaching approach. However, a notable subset of students did not share this sentiment.

Notably, as 2022 marked the third year of the COVID-19 pandemic, during which students encountered hybrid teaching across various subjects, the initial enthusiasm waned and the cumulative pressure from multiple subjects became evident. This trend was reflected in the data, indicating a decrease in the perceived benefits of hybrid teaching.

Empirical evidence from our study suggests that the perception of hybrid teaching’s effectiveness directly correlates with students’ preferences for educational methods. Students who perceived hybrid teaching as beneficial overwhelmingly preferred this mode of instruction, while those who did not perceive benefits were more likely to prefer offline teaching. This underscores the critical role of learning ability enhancement in shaping students’ educational preferences and the need to continuously refine our teaching strategies to meet these evolving needs.

### Future directions for research

While the current study provides valuable insights into hybrid teaching methodologies and their effectiveness, there is ample scope for further exploration, particularly in the post-COVID-19 era. Given the pivotal role of hybrid teaching in teaching reform, enhancing students’ acceptance and recognition of this approach is paramount. Future research endeavors should build upon the existing findings by exploring the following avenues:

1. Curriculum Reorganization: Our data suggests that students’ preferences for hybrid learning methods align with a curriculum that incorporates micro-lectures, recorded presentations, and interactive learning activities. Future studies could investigate the effect of reorganizing curricula to prioritize the integration of basic and clinical knowledge, as well as the optimal utilization of school network resources, such as renowned faculty lectures and virtual simulation classrooms. The effectiveness of these changes can be assessed through student performance and feedback analysis.
2. Development of an Online Teaching Library: Based on the hybrid teaching modality, future research could focus on constructing a comprehensive online teaching library. This library should include basic resources (e.g., course outlines, key concepts, and teaching objectives), advanced resources (e.g., online case studies, clinical simulations, and group projects), and evaluative resources (e.g., pre- and post-lecture assessments). The effectiveness of the library can be determined by analyzing the correlation between student engagement with the resources and their academic performance.
3. Cultivation of Medical Innovation Talents: In accordance with the objectives of education reform, future research endeavors to cultivate individuals endowed with a profound knowledge foundation, significant potential for innovation, and robust clinical capabilities. Given that active learning can elevate academic performance and decrease dropout rates [21], a potential area of research could be to construct an online platform showcasing exemplary student work, such as presentations, mind maps, and educational videos, on stimulating peer engagement and collaborative learning.
4. Integration of Emerging Technologies: The potential role of emerging technologies, such as game-based learning [22], simulation tools, and virtual reality [23], in alleviating psychological pressures and fostering interactive learning environments merits further investigation. Additionally, the utilization of artificial intelligence (AI) tools, exemplified by platforms such as ChatGPT [24, 25] could introduce novel opportunities for refining both the teaching and learning processes.

## Conclusions

The present study is focused on examining whether students favor hybrid teaching methodologies and suggesting ways to refine teaching techniques and amplify their overall efficacy. Hybrid teaching method has proven to be an effective teaching model, particularly in enhancing the effectiveness of Physiology teaching. However, over the past four years, a number of challenges have been encountered and require urgent attention to be solved. Nevertheless, there remains much room for improvement and further exploration to enhance our teaching methods in the future. It is, therefore, our ongoing task to identify and address these issues as we strive to optimize the hybrid teaching experience.

### Electronic supplementary material

Below is the link to the electronic supplementary material.

Supplementary Material 1

Supplementary Material 2

Supplementary Material 3

Supplementary Material 4

## Data Availability

Data is provided within the supplementary information files.
