# Supplementary material for: Hybrid teaching after COVID-19: advantages, challenges and optimization strategies
Source: BMC Med Educ. 2024 Jul 12;24:753. doi: 10.1186/s12909-024-05745-z (PMC11241882; doi:10.1186/s12909-024-05745-z)
Supplement: Supplementary file 3 — Supplementary Material 3 [file 12909_2024_5745_MOESM3_ESM.docx]

**Hybrid teaching after COVID-19: Advantages, challenges and optimization strategies**

Xiaoran Wang^1^#, Jiangheng Liu^2^#, Shuwei Jia^1^#, Chunmei Hou^1^, Runsheng Jiao^1^, Yan Yan^1^, Tengchuang Ma^2^, Ying Zhang^1^, Yanyan Liu^1^, Haixia Wen^1^,Yu-Feng Wang^3^, Hui Zhu^1^*, and Xiao-Yu Liu^1^*

Supplemental file 3. Example of questionnaire for hybrid teaching method in Physiology

1. Your gender is

A, male

B, female

2. Your major is

A, four years

B, five years

C, seven years

D, eight years

3. (Multiple choices) What do you think are the advantages or benefits of current online teaching in Physiology?

A, Increase of information gain

B, Clear learning objectives

C, Conducive to resource sharing

D, Convenient for interaction and communication between teachers and students

E, Help to broaden the horizon

F, Record the screen of teaching content

4. (Multiple choices) What do you think are the disadvantages of current online teaching methods in Physiology?

A, Too much information for understanding;

B, The teaching speed is too fast to follow;

C, It took too much time in autonomic learning.

D, Unable to communicate with teachers face to face;

E, It is difficult for students to judge whether resources are good or bad.

5. Among various online teaching methods, which do you like best

A, Live class

B, Recorded lecture

C, Microlecture + interaction

D, Microlecture + Recorded lecture + interaction

6. Do you think that the hybrid teaching helped improve your learning ability?

A, Great improved

B, Same with usual

C, A little help

7. How much time do you spend in your autonomous learning in Physiology?

A, 10 ~ 30 min

B, 30 ~ 60 min

C, 1 ~ 2 h

D, more than 2 h

8. Does the hybrid teaching in Physiology increase your learning burden?

A, Strongly increased

B, Increased

C, Fair

D, Reduced
