# Supplementary material for: Hybrid teaching after COVID-19: advantages, challenges and optimization strategies
Source: BMC Med Educ. 2024 Jul 12;24:753. doi: 10.1186/s12909-024-05745-z (PMC11241882; doi:10.1186/s12909-024-05745-z)
Supplement: Supplementary file 4 — Supplementary Material 4 [file 12909_2024_5745_MOESM4_ESM.docx]

**Hybrid teaching after COVID-19: Advantages, challenges and optimization strategies**

Xiaoran Wang^1^#, Jiangheng Liu^2^#, Shuwei Jia^1^#, Chunmei Hou^1^, Runsheng Jiao^1^, Yan Yan^1^, Tengchuang Ma^2^, Ying Zhang^1^, Yanyan Liu^1^, Haixia Wen^1^,Yu-Feng Wang^3^, Hui Zhu^1^*, and Xiao-Yu Liu^1^*

Supplemental file 4. Course structures of the HBOPPPS

| **Main contents** | **Time** | **Way of presentation** |
| --- | --- | --- |
| Bridge in | Before lecture | **Online:** discussions, expanded knowledge, science stories, micro-video lecture |
| Activity in the classroom | 90 min |  |
| Check attendance | At the beginning of lecture | **Online** |
| Objective and expected outcomes | 2 min | **Online and PPT:** show course introduction and what students will gain from the course |
| Pre-assessment | 3 min | **Online or Offline:** one multiple choice-single answer questions (Mobile App) |
| Participatory learning | 75 min | **Offline:** PPT, lectures, handwriting on the blackboard, implication questions, group discussion, dynamic pictures, videos, raise random questions, mobile phone shake to select a student randomly (Mobile App) |
| Post- assessment | 5 min | **Online:** 3~5 multiple choice-single answer questions (Mobile App) |
| Summary | 5 min | **Offline:** Summarize the key contents based on blackboard writing with emphasis marks and associated figures in PPT |
| Mind map or topics | After lecture | **Online:** post online for student-led summary and discussion |
| Post-lecture interactions (mentoring) | After lecture | **Online:** posting questions, private chat with teachers, provide feedback information |
